# Doing without codeine: why and what are the alternatives?

**DOI:** 10.1186/1824-7288-40-16

**Published:** 2014-02-11

**Authors:** Franca Benini, Egidio Barbi

**Affiliations:** 1 Pediatric Pain and Palliative Care Service, Department of Maternal and Child Health, University of Padua, Padua, Italy; 2 Institute for Maternal and Child Health-IRCCS “Burlo Garofolo”, Trieste, Italy

**Keywords:** Codeine, Pain, Child, Side effect

## Abstract

Codeine is a mild opioid widely used as an analgesic in various age groups, including various pediatric settings. It is a prodrug that owes its analgesic effect almost entirely to the principal metabolite: morphine. The genetic polymorphisms can contribute to making the pharmacokinetics of codeine hard to predict and this it is particularly important in the pediatric population because infants and children have greater susceptibility to the side-effects of morphine. In recent years there have been several reports in the literature on the risks relating to the use of codeine. In August 2012, the American Food and Drugs Administration began to revise its recommendations for the safe use of codeine and in February 2013, established that codeine should not be used for postoperative pain control in children undergoing adenoidectomy and/or tonsillectomy and did restrict the use of this drug in the pediatric population. In June 2013, the European Medicine Agency opted the same decision. In July 2013, the Agenzia Italiana del Farmaco prohibit the use of medicines containing codeine for patients under 12 years old and recommended a limited use of the drug, in many other situations. Complying with these recommendations naturally means changing habits and treatment strategies well established in pediatric practice, but other drugs, tools and techniques available enable us to continue to assure an adequate pain control in pediatric patients, irrespective of their age and situation. The article proposes same alternatives of pain control drugs.

## Background

Codeine is a mild opioid widely used as an analgesic in various age groups, including various pediatric settings.

It is a prodrug that owes its analgesic effect almost entirely to the principal metabolite: morphine. Cytochrome P450 2D6 (CYP2D6) converts codeine into morphine in the liver. This process is genetically determined and the enzyme’s activity varies from one person to another. The enzyme is more active in some individuals (termed *ultra-rapid metabolizers*), converting codeine into morphine more quickly and completely. This also means that, after they have been administered the right dose of the drug for their ideal weight and age, these individuals have higher blood levels of morphine than controls. The prevalence of this mutation varies among different ethnic groups, ranging from 5% to 29%. Meanwhile, a variable proportion of 5-10% of the population has a limited CYP2D6 enzymatic activity (*poor metabolizers*), in which case the amount of morphine produced by their metabolism of codeine is very small.

Like the polymorphism of cytochrome 450 isoenzyme CYP2D6, other genetic polymorphisms can also contribute to making the pharmacokinetics of codeine hard to predict (including variations in morphine metabolism, its passage through the blood–brain barrier, and different receptor kinetics). All these genetic variations can have clinical consequences, and an unfortunate combination of different polymorphisms can give rise to toxic levels of morphine - even after an appropriate dose of codeine has been administered - with a higher risk of side-effects.

*This is particularly important in the pediatric population because the fact of being newborn or an infant is associated per se with a greater susceptibility to the side-effects of morphine*[1,2].

## Main text

In recent years there have been several reports in the literature on the risks relating to the use of codeine.

In 2007 Madadi [3] reported on the death of a newborn breastfed by a mother who had been given codeine to control postpartum pain. An analysis of the mother’s genotype revealed that she was an ultra-rapid metabolizer and the newborn’s blood contained toxic levels of morphine.

Ciszkowski reported in 2009 [4] on a two-year-old child who died after being sent home on codeine therapy (at the correct dose) to control postoperative pain after an adenotonsillectomy. The autopsy showed that the infant had pneumonia and high blood levels of morphine. Genotyping confirmed that the child was a hyper-metabolizer. The author made the point that, combined with the high morphine levels, other factors may have contributed to the fatal outcome, i.e. pneumonia and sleep apnea may have exacerbated the infant’s hypoxemia, consequently altering the mu receptors and increasing their sensitivity to morphine.

Kelly et al. [5] reported on 3 fatal cases of respiratory depression and 1 of severe respiratory insufficiency occurring between 2009 and 2012 in children between two and five years old. They were all ultra-rapid metabolizers who had been given codeine after tonsillectomy and/or adenoidectomy for obstructive sleep apnea syndrome [5]. Here again, these patients had several risk factors, i.e. they were young children, they had obstructive sleep apnea, they had undergone surgery, and they were also ultra-rapid CYP2D6 metabolizers.

Niester et al. [6] conducted a literature review on the cases of respiratory depression due to opioids in a pediatric setting (0 to 12 years old). Among 24 reports, they identified 27 cases of severe respiratory depression induced by opioids, 7 of which were fatal. Eight cases were due to an overdosage of the drug; for the other 19, three specific causes were identified: opioid accumulation due to a renal insufficiency that had been overlooked; patients who were hyper-metabolizers; and codeine therapy used for patients who had tonsillectomy and/or adenoidectomy for recurrent obstructive sleep apnea or tonsillitis (and whose respiratory depression probably coincided with a hypoxia that exacerbated their susceptibility to a drug-related respiratory depression.

In 2013, Friedrichsdorf [7] described three cases of children ranging from four to 10 years old dying at home due to codeine toxicity. All three were overweight or obese, but the prescribed dose of the drug was appropriate for their ideal weight. In this article, obesity and polypharmacology were mentioned as concomitant causes of toxicity.

In August 2012, the American *Food and Drugs Administration (FDA)* began to revise its recommendations for the safe use of codeine. From 1969 to 1 May 2012, there were 13 reported cases of adverse events or death in patients on codeine therapy (10 deaths and 3 cases of severe respiratory insufficiency), in patients ranging from 21 months to 9 years of age. Most of these reports concerned children with obstructive apnea who had been given appropriate doses of the analgesic for postoperative pain control after tonsillectomy and/or adenoidectomy (8 cases), or during the course of respiratory infections (3 cases). Among the 7 cases in which a genetic study on cytochrome P450 2D6 was conducted, an increased enzyme activity was always documented: 3 were “ultra-rapid metabolizers”, 1 was a “probable ultra-rapid metabolizer”, and 3 were “extensive metabolizers”. All these patients had respiratory problems, which were judged to be factors capable of increasing these young patients’ susceptibility to the respiratory depressant effects of toxic levels of morphine [8-10].

Since it is impossible to identify children who are ultra-rapid metabolizers of codeine in advance, and given the correlated risks (albeit only in rare reports), the FDA established that codeine should not be used for postoperative pain control in children undergoing adenoidectomy and/or tonsillectomy. While the FDA did not restrict the use of codeine for pain control in other clinical situations, it did reiterate the need to monitor patients for the drug’s efficacy and side-effects (particularly sleepiness, sedation, breathing rate and characteristics) continuously during the treatment.

In February 2013, the FDA reported on another 3 deaths and 1 case of severe respiratory insufficiency in young patients treated with codeine. All the children involved were ultra-rapid or extensive metabolizers, they developed high blood levels of morphine, and they were given codeine therapy to control postoperative pain after tonsillectomy and/or adenoidectomy [11]. On this occasion, the FDA confirmed its ban on the use of codeine in children undergoing surgery for tonsillectomy and/or adenoidectomy for obstructive apnea. It also stated that, in the event of drugs containing codeine being used for other conditions, they should be prescribed at the lowest effective dose, for the shortest possible period of time, and only administered as necessary, not according to any fixed schedule.

In October 2012, the *European Medicine Agency (EMA)* also began a review procedure on the risk-benefit profile of products containing codeine used for pain control in pediatric patients. At the end of the review process, completed in mid-2013 (EMA, 28 June 2013), the EMA opted to prohibit the use of medicines containing codeine for patients under 12 years old, and extended this ban up to 18 years of age in the case of patients undergoing tonsillectomy and/or adenoidectomy for obstructive sleep apnea. It also recommended that, when used, these drugs should be prescribed at the minimum effective dose, for the shortest possible period of time, with continuous monitoring of their efficacy and side-effects [12].

On 29 July 2013, the *Agenzia Italiana del Farmaco (AIFA)* prohibited the use of codeine for pain control in certain categories of patients, i.e. [13]:

•children under 12 years of age,

•children and adolescents (0–18 years old) undergoing adeno/tonsillectomy for obstructive sleep apnea syndrome,

•patients known to be hyper-metabolizers, and

•breastfeeding women.

It also recommended that products containing codeine be avoided for children over 12 with any respiratory function impairment, and it reiterated the EMA considerations on the dosage and duration of the treatment.

In the light of the above, it is worth adding some comments and conclusions:

•*The reported adverse events are very rare and multifactorial, but catastrophic in their evolution.*

•*The feasibility of performing diagnostics to predict which might become critical is very limited and uncertain.*

•*Monitoring side-effects may help to contain the negative evolution of toxicity episodes, but cannot prevent their onset.*

•*The literature available on the pharmacokinetics, pharmacodynamics and toxicity of codeine is dated, limited, and does not allow for any final conclusions to be drawn.*

•*Codeine is scarcely or not at all effective in some children who carry the cytochrome 450 isoenzyme CYP2D6 polymorphism.*

•*Other analgesics are available for use in the pediatric setting as an alternative to codeine.*

Hence the recommendations of the FDA, EMA and AIFA, and it is important to bear these protective measures in mind when deciding whether or not to use codeine.

Complying with these recommendations naturally means changing habits and treatment strategies well established in pediatric practice, but other drugs, tools and techniques available enable us to continue to assure an adequate pain control in pediatric patients, irrespective of their age and situation.

Recent data have demonstrated that, among the non-opioids, there are drugs that are very well known, easy and safe to use but that continue even today to be little used in children (and often in insufficient doses). This is the case of paracetamol and ibuprofen, for example, which are indicated for a great deal of situations requiring mild-to-moderate pain control in pediatric age [14].

Among the mild opioids, *tramadol* is the best alternative to codeine in the pediatric setting*.* Tramadol is a synthetic 4-phenylpiperidine codeine analog. Its analgesic potency is considered to be medium, acting on both opioid-sensitive and opioid insensitive pain through multiple mechanisms of action. It is considered a weak mu-opioid receptor agonist. It inhibits the neuronal reuptake of serotonin and norepinephrine. It displaces serotonin stores within the spinal cord, facilitating the descending pain inhibitory pathways. It may have selective spinal and local anesthetic effects on peripheral nerves [15]. The opioid activity of tramadol results from a low affinity for binding to mu-opioid receptors with no affinity for delta or kappa opioid receptors. The drug is bio-activated by CYP2D6 to the opioid receptor agonist O-desmethyltramadol, which contributes to the analgesic effect because of its 300-fold higher affinity for binding to mu-opioid receptors [15,16].

The analgesic efficacy and safety of tramadol in children have been confirmed by various studies in different settings, in both inpatients and outpatients, including cases of adenotonsillectomy in children with obstructive sleep apnea [15,17-25].

The advantages of tramadol over opioids lie mainly in the lower incidence of major side effects such as ventilatory depression and sedation. An ample, up-to-date literature search brought to light no reports of any respiratory depression or sedation in the pediatric age group. On the other hand, dose-dependent hypercapnia was reported in one group of neonates, suggesting that tramadol affects the respiratory center during the neonatal period [26].

To the best of our knowledge, there are only three case reports in the literature of tramadol at therapeutic doses inducing respiratory depression in adults: one of these involved an CYP2D6 ultra-metabolizer patient with renal impairment; the other two cases are controversial, occurring in patients concomitantly using phentanyl and other anesthetics [27-29].

Nausea-vomiting, pruritus and rash are the most frequently reported side-effects of tramadol, occurring with a frequency of around 10%, 7% and 4%, respectively [30].

The importance of pharmacogenetics has been highlighted when it comes to considering the risks of tramadol causing adverse effects: CYP2D6 gene duplication carriers are at a statistically significant higher risk of developing adverse events (mainly vomiting and nausea), without any pharmacokinetic differences. The proportion of the population with this gene duplication is reportedly quite high in southern Europe and northern Africa (up to 7% in Spain and Turkey, 30% in Ethiopia and Saudi Arabia) [31].

In Italy, tramadol is licensed for use in children over 1 year old.

In conclusion, judging from the literature, tramadol seems to be safe for use in children, be they inpatients or outpatients. Given the lessons learned from the codeine-related deaths, however, we should bear in mind that specific risk factors (adeno-tonsillectomy, sleep apnea, obesity, renal impairments, the neonatal period) could have a role in any onset of adverse events in ultra-metabolizers. The body of evidence currently available is still too limited to thoroughly ascertain tramadol’s safety in such cases, however. In children with specific risk factors, it may therefore be appropriate to limit the use of tramadol to monitored settings while we wait for more data to emerge.

In cases of moderate to severe pain, strong opioids (especially morphine and oxycodone) provide a valid and appropriate alternative to codeine. Used correctly, these drugs are effective, manageable and very safe [14].

## Conclusion

The limitations imposed on the use of codeine in pediatric practice should not have the wholly unjustified effect of triggering or exacerbating concern or fears about prescribing analgesic drugs in general, and opioids in particular, for children. They should contribute instead to an increasing our awareness, attention to patients’ symptoms, and competent, critical analysis of the many options currently available for pain control.

All newborn, infants and children have a right to pain control, and the appropriate tools for achieving this aim are available to all health-care professionals.

## Abbreviations

CYP2D6: Cytochrome P450; FDA: Food and Drugs Administration; EMA: European Medicine Agency; AIFA: Agenzia Italiana del Farmaco

## Competing interests

Authors declare that they don’t have financial and/or non financial competing interest, in this article.
